# Effect of Xuefu Zhuyu Decoction Pretreatment on Myocardium in Sepsis Rats

**DOI:** 10.1155/2018/2939307

**Published:** 2018-09-09

**Authors:** Fansu Meng, Haibiao Lai, Zekun Luo, Yong Liu, Xiaoxun Huang, Junqian Chen, Bayi Liu, Yingjun Guo, Yu Cai, Qingqing Huang

**Affiliations:** ^1^Zhongshan Hospital of Traditional Chinese Medicine Affiliated to Guangzhou University of TCM, Zhongshan, Guangdong 528400, China; ^2^Guangdong Provincial Hospital of Chinese Medicine, The Second Affiliated Hospital of Guangzhou University of Chinese Medicine and Guangdong Provincial Academy of Chinese Medical Sciences, Guangzhou 510006, China; ^3^College of Pharmacy, Jinan University, Guangzhou 510632, China

## Abstract

Xuefu Zhuyu Decoction (XFZYD), the classical recipe for promoting blood circulation by removing blood stasis, has been used in China for a long history clinically. XFZYD has been found to improve cardiac function through reducing inflammation. However, the effect of XFZYD on myocardial apoptosis remains unclear. Herein, we investigated the mechanism of XFZYD preconditioning on myocardial injury in sepsis rats. The rats were treated with XFZYD one week, followed with intraperitoneal injection of lipopolysaccharide (LPS: 10 mg/kg) to induce sepsis. Pretreatment with XFZYD could reverse the effects of LPS-induced decreased mean arterial pressure (MAP) and increased heart rate (HR). XFZYD decreased the levels of malondialdehyde (MDA), superoxide dismutase (SOD), tumor necrosis factor-*α* (TNF-*α*), interleukin-1*β* (IL-1*β*), and interleukin-6 (IL-6) in serum or in heart. TUNEL staining revealed that the apoptotic index of XFZYD was significantly lower compared with the LPS group (P<0.05). Western blot results showed that the high doses of pretreatment XFZYD group can reduce the Bax expression of myocardial tissue in rats (P<0.05, P<0.01). The expression of Bcl-2 in XFZYD group was significantly higher than that in the LPS group (P<0.01), while the expression of caspase-3 in treatment group was significantly lower than that in the LPS group only after 12 h modeling (P<0.01). In addition, caspase-3 activity in rat cardiomyocytes of XFZYD-treated animals was significantly decreased. These findings suggest that pretreatment with XFZYD exerts a protective effect in the myocardium of septic rats by inhibiting myocardial cell apoptosis and antioxidation.

## 1. Introduction

Sepsis, which is a critical illness causing mortality all over the word, was defined as “life-threatening organ dysfunction due to a dysregulated host response to infection” by the Third International Consensus Definitions Task Force [1, 2]. Excessive production of cytokines induced by infectious stimuli is a marker of sepsis, leading to multiple organ dysfunction and, consequently, high mortality [3]. Myocardial dysfunction is a recognized manifestation of this fatal disease [4–6]. Additionally, inflammatory responses are also of great importance during sepsis [7]. LPS is the main molecular component of the outer membrane of Gram-negative bacteria and acts as a physical barrier to provide bacterial protection for the surrounding environment [8]. However, toxic LPS often leads to a variety of biological effects ranging from significantly increased anti-infective to uncontrolled and massive immune response leading to sepsis and septic shock [9].

Many candidate pathophysiological mechanisms may be involved in the induction of myocardial dysfunction in septicemia. Respectively, apoptotic-inflammatory imbalance is important molecular mechanism underpinning the development of sepsis [10]. It has been observed that cardiac function decreased and myocardial TNF-*α* and IL-6 levels increased whereas TLR-4 knockout mice had attenuated responses to lipopolysaccharide challenge in a septic model [11]. Caspase-8 can be activated by TNF and then activates caspase-3—a crucial apoptotic protease in the final common pathway of the apoptotic cell-death programme [10]. Moreover, it is important to note that sepsis decreases the level of expression of BCL-2 and increases in Bax expression which contributes to apoptosis [12, 13]. Thus, these potential factors that are responsible for sepsis allow for the development of targeted therapy.

XFZYD, a traditional Chinese herbal formula including 11 herbs (Table 1), is first recorded in a book called* Yi Lin Gai Cuo* by Wang Qingren [14]. It is believed that XFZYD could activate blood and resolve stasis for treating cardiovascular diseases in Chinese medical theory [15]. Previous studies have shown that XFZYD can significantly reduce TNF-*α* content in the serum of rabbits [16]. Some studies have shown that XFZYD can interfere with cardiomyocyte apoptosis in vitro [17], but it has not been reported in vivo and the effect of XFZYD on myocardium remains unclear. The purpose of this study was to analyze the effect of preadministered XFZYD on myocardium in rats with lipopolysaccharide-induced sepsis and to elucidate the mechanism of XFZYD preconditioning on cardiomyocytes in sepsis rats.

## 2. Materials and Methods

### 2.1. Chemicals and Reagents

*Prunus persica, Angelicae sinensis*,* Ligusticumi chuangxiong, Carthamus tinctorius, Paeonia lactiflora*,* Rehmannia glutinosa*,* Citrus aurantium*,* Bupleurum Chinese*,* Platycodon grandiflorum*,* Achyranthes bidentata*, and* Glycyrrhiza uralensis* are provided by Fangcun Taizhen Pharmacy in Guangzhou. All Chinese medicines were identified by Professor Zhiguo Ma, who engaged in the study of traditional Chinese medicines. All materials were appraised according to the Pharmacopoeia of the People's Republic of China (2015). LPS (LPS,* E. coli* serotype 055:B5) was purchased from Sigma-Aldrich (St. Louis, MO, USA). MDA Assay Kit, SOD Assay Kit, and BCA Protein Assay Kit were purchased from Nanjing Jiancheng Bioengineering Institute (Jiangsu, China); TUNEL Assay Kit (POD) was obtained from Roche (USA); Rat TNF-alpha Quantikine ELISA Kit was purchased from R&D Systems (USA); caspase-3 activity test kit was obtained from Biovision (USA); HRP-labeled goat anti-rabbit G secondary antibody, Bcl-2 rabbit anti-mouse mAb, and Bax rabbit anti-mouse monoclonal antibody were purchased from Abcam (USA); caspase-3 rabbit anti-mouse monoclonal antibody was obtained from cell signaling (USA); DAB working fluid and hematoxylin were purchased from Biyuntian Institute of Biotechnology (Jiangsu, China).

### 2.2. Animals

120 male Sprague Dawley rats (250±20g) were provided by Jinan Pengyue Experimental Animal Breeding Co., Ltd., license number: SCXK (Lu) 20140007. All animals care and experiments were performed under institutional protocols approved by the Institutional Animal Care and Use Committee at Jinan University, Guangzhou, China. The animals were kept under standard conditions (free access to food and water, temperature 25°C, 12 h/12h light/dark cycles).

### 2.3. Preparation and Quality Control of Xuefu Zhuyu Decoction

**Figure 1:**
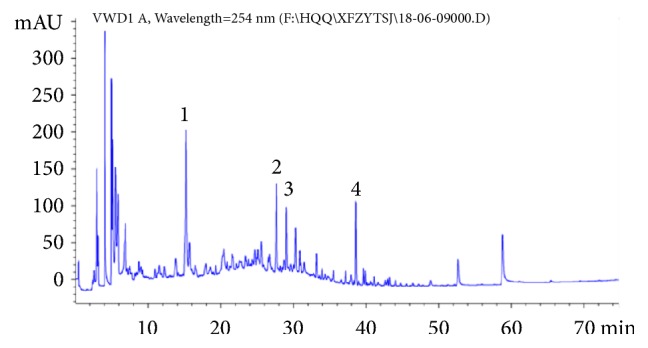
The HPLC chromatogram of XFZYD. Four major compounds (1-4) were identified and compared to the standards. Peak number: 1 Amygdalin, 2 naringin, 3 paeoniflorin, and 4 ammonium glycyrrhizinate.

After soaking for 1 h, the XFZYD were prepared by water extraction twice. The extract was filtered and condensed to make the concentration equivalent to 9.0 g herb/mL and then kept at 4°C. To control the XFZYD quality, high performance liquid chromatography (HPLC) method was performed to establish the fingerprint spectrum. The chromatographic profile of the XFZYD is shown in Figure 1. The analyses were performed with a Agilent 1260 system (Agilent Technologies, Hewlett-Packard-strasse-8, 76337 Waldbronn, Germany) equipped with a quaternary gradient pump. The components were eluted with a gradient system consisting of water (A) and acetonitrile (B). The elution gradient was 0-15 min (B: 5%-15%), 15-25 min (B: 15%-25%), 25-40 min (B: 25%-50%), 40-60 min (B: 50%-75%), 60-75 min (B: 75%-95%). The chromatographic column was Agilent ZORBA× SB-48 (4.6 × 250 mm, 5 *μ*m). The mobile phase flow rate was 1 mL/min and column temperature was maintained at 25±1°C. The detection wavelength was 254 nm with injection volume of 20 microliters. Otherwise, Amygdalin, naringin, paeoniflorin, and ammonium glycyrrhizinate were dissolved in methanol as control detected by the HPLC method.

### 2.4. Animal Experiments

After 7 days of adaptive feeding, rats were randomly divided into 5 groups: sham (saline, n=20), LPS (10mg/kg, n=20), LPS+XFZYD (3.9g/kg, n=20), LPS+XFZYD (7.8g/kg, n=20), and LPS+XFZYD (15.6g/kg, n=20). The normal sham group served as negative control given saline one week and was not exposed to LPS, while remaining rats were intraperitoneally injected with LPS after one-week treatment by gavage. 12 h or 24 h after treated with LPS, the average blood pressure and heart rate of rats were measured via tail cuff plethysmography (IITC Life Science MRBP Blood Pressure System, California, USA).Then the heart tissues and plasma were taken at the 12th and 24th hours after LPS administration.

### 2.5. Specimen Collection and Preservation

Animals from each group were anesthetized with 0.1% (vol/vol) pentobarbital sodium (4mL/kg). The rat heart was harvested, and 2 pieces of myocardial tissue of each group from different time point were selected from the front wall of the left ventricle after being flushed with iced-cold salt water. Each tissue sample was fixed with 10% (vol/vol) formaldehyde, which was then embedded and sliced for H&E staining. The remaining myocardial tissue was stored at −80°C. The abdominal aorta blood (5 mL) was collected and centrifuged at 4°C 3000 rpm for 15 minutes. Then the supernatant was stored at −70°C to be tested.

### 2.6. Hematoxylin-Eosin (H&E) Staining

At the end of the experiment, the hearts (apical apex) were excised and fixed with neutral 10% formalin, dehydrated, embedded in liquid paraffin, and stained with H&E. Finally the myocardial cell morphological changes were observed under an optical microscope.

### 2.7. TUNEL Labeling Examination

Myocardial apoptosis was analyzed using a terminal deoxynucleotidyl transferase UTP nick end labeling (TUNEL) assay. The paraffin-embedded tissue was cut into sections 5 *μ*m thick. The tissue sections were deparaffinized into water and incubated with protease K (20 *μ*g/ml) at 37°C for 30 min. Then TUNEL reaction mixture (50*μ*L) was added to each sample and the slides were incubated in a humidified atmosphere for 60 min at 37°C in the dark. Slides were rinsed with phosphate buffered saline (PBS; PH 7.4) three times, for 5 min each time. After the slides were dried, 50 *μ*L converter-POD was added to the samples, and coverslips were incubated in a wet box at 37°C for 30 minutes. To detect the nuclei, slides were incubated with DAB for 10 min at room temperature in the dark and observed under a light microscope. According to the distribution of apoptotic cells, four 400× high-power fields were taken from each slice, and 250 cells were counted in each field and expressed as percentages as apoptotic index (AI),

(1) $$\begin{array}{c} AI=apoptosis-\frac{positive\;\;cells}{the\;\;total\;\;number\;\;of\;\;cells}\times 100\%. \end{array}$$

### 2.8. Detection of MDA Content and SOD Activity in Myocardial Tissue

Weigh the appropriate amount of tissue and add 9 volumes of normal saline according to the weight (g): volume (mL) = 1:9 ratio. Then the homogenate was prepared in an ice water bath and centrifuged at 3000rmp/min for 10 minutes. The supernatants were extracted to quantified protein concentration using bicinchoninic acid (BCA) method and further assay. Finally, the MDA content and SOD activity of myocardial tissue were detected according to the kit instructions.

### 2.9. Determination of TNF-*α*, IL-*β*, and IL-6

Blood samples were collected from the abdominal aorta and centrifuged at 3000 rpm for 10 minutes to obtain serum. Myocardial tissue and serum stored at −80°C. Then the levels of TNF-*α*, IL-*β*, and IL-6 were measured according to the ELISA kit instructions.

### 2.10. Western Blotting

Myocardial tissue (100 mg) was placed in precooled cell lysate (1mL) and tissue homogenates were prepared by homogenizer. Protein quantification was performed using the BCA method. Equal amounts of total protein (30*μ*g) were separated on 10% SDS-PAGE. Then the proteins were transferred onto nitrocellulose membranes (EMD Millipore, Billerica, MA, USA) and incubated in 5% skimmed milk for 2 hours. The membrane was washed for 5 min with 1x PBST, total of 3 times. The membrane was placed in the primary antibody against Bcl-2, Bax, or caspase-3 (1:2000) and incubated overnight at 4°C. *β*-actin was selected as the loading control. Membranes were washed 5 times with PBST for 5 minutes and incubated with secondary antibody solution (1:5000) at room temperature for 1 h, and then washed with PBST. Immunoreactive bands were imaged using a Tanon-5200 imaging system and quantified using the Image J software.

### 2.11. Cardiomyocyte Caspase-3 Activity Assay

Caspase-3 activity was measured by caspase-3/CPP32 Colorimetric Assay (BioVision). In brief, the heart tissues were homogenized and centrifuged at 12000 rpm for 10 min at 4°C. Then the supernatants were extracted to quantified protein concentration using BCA method and further assayed for caspase-3 activity. 100 ug of protein was diluted to 50 uL of cell lysis buffer per assay and added 50 uL of 2X reaction buffer (10 mM DTT). Then the samples were incubated with 5uL of 4 mM DEVD-PNA substrate (200uM final concentration) for 2 hours at 37°C. The samples without the DEVD-pNA substrate were zeroed and colorimetrically read at 405 nm in a microtiter plate reader. The results are expressed as OD405.

### 2.12. Statistical Analysis

Statistical analysis was performed using GraphPad Prism 6 software. Data was expressed as mean ± standard. Multiple-sample comparisons were performed using one-way ANOVA, with a statistical difference at p<0.05.

## 3. Results

### 3.1. HPLC Analysis

To investigate the quality of the XFZYD, four major compounds were used as quality control standards for XFZYD. The HPLC chromatogram of XFZYD was achieved on a C18 column using a mixture of acetonitrile and water with 0.1% formic acid as the mobile phase. The HPLC chemical fingerprint of XFZYD was shown as in Figure 1. The four major compounds were (1) Amygdalin, (2) naringin, (3) paeoniflorin, and (4) ammonium glycyrrhizinate, respectively.

### 3.2. Pretreatment of XFZYD on LPS-Induced MAP and HR

As shown in Table 2, administration of LPS resulted in a significant decrease in MAP and a significant increase in HR (*P <* 0.05). High dose of XFZYD could relieve the LPS-induced decrease of MAP. Also, XFZYD significantly moderated the LPS-induced increase of HR at both 12 h and 24 h.

### 3.3. H&E Staining

**Figure 2:**
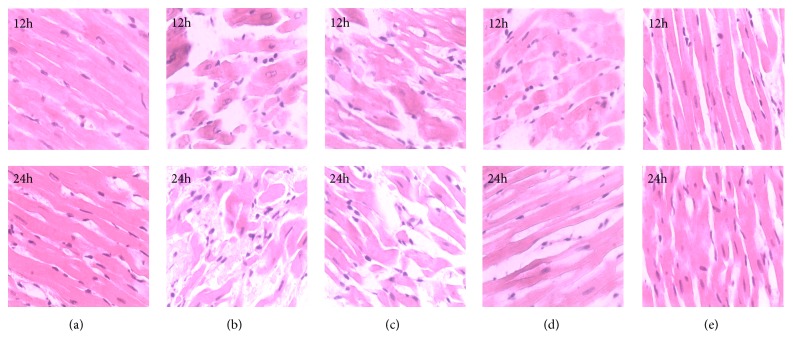
H&E staining of myocardial tissue in sepsis rats (magnification × 400; modeling 12 h and 24 h). (a) Saline group; (b) LPS (10mg/kg) group; (c) LPS+XFZYD (3.9 g/kg); (d) LPS+XFZYD (7.8 g/kg); (e) LPS+XFZYD (15.6g/kg).

As shown in Figure 2, there was no vasodilation or inflammatory cell infiltration in the sham group, while extensive inflammatory cells infiltrated tissues and a large number of mononuclear cells mixed with a few lymphocytes and neutrophils in the LPS group. The degree of myocardial damage in the low-dose XFZYD group was reduced; however, the myocardial structure still remained damaged including inflammatory cell infiltration, hemorrhage, and cell necrosis (Figure 2(c)). By contrast, the conditions of middle and high dose XFZYD groups were much better than those in LPS or low-dose XFZYD groups (Figures 2(d) and 2(e)). These results show that XFZYD may reduce myocardial damage and play a protective role in the heart structure and function of septic shock rats.

### 3.4. Apoptotic Cell Detection

**Figure 3:**
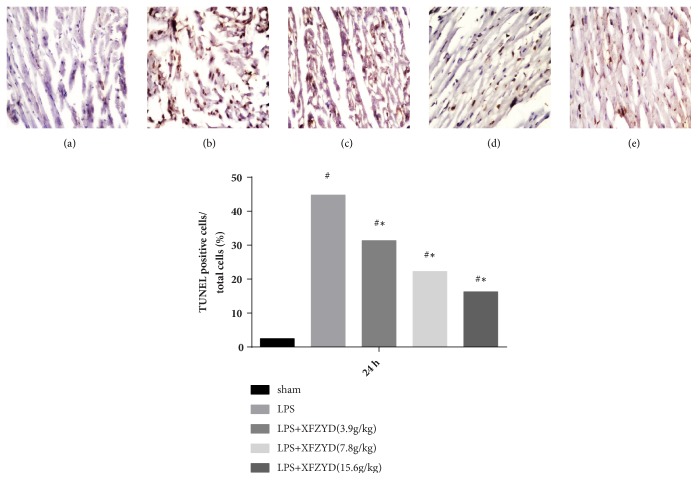
LPS-induced myocardial apoptosis of 24 h with different pretreatment. Apoptotic cells were detected by TUNEL assay kit. Representative images are shown (magnification, ×400). Data are presented as the mean±standard error, n=6 per group. # p <0.01 versus sham group; *∗* P <0.01 versus LPS group.

The number of TUNEL-positive cells detected in myocardial tissue was increased in the LPS group compared with the sham group (Figure 3), indicating a significantly higher degree of apoptosis. The apoptotic index was significantly lower in the XFZYD pretreatment group compared with the LPS group.

### 3.5. Detection of MDA Content and SOD Activity in Myocardial Tissue

**Figure 4:**
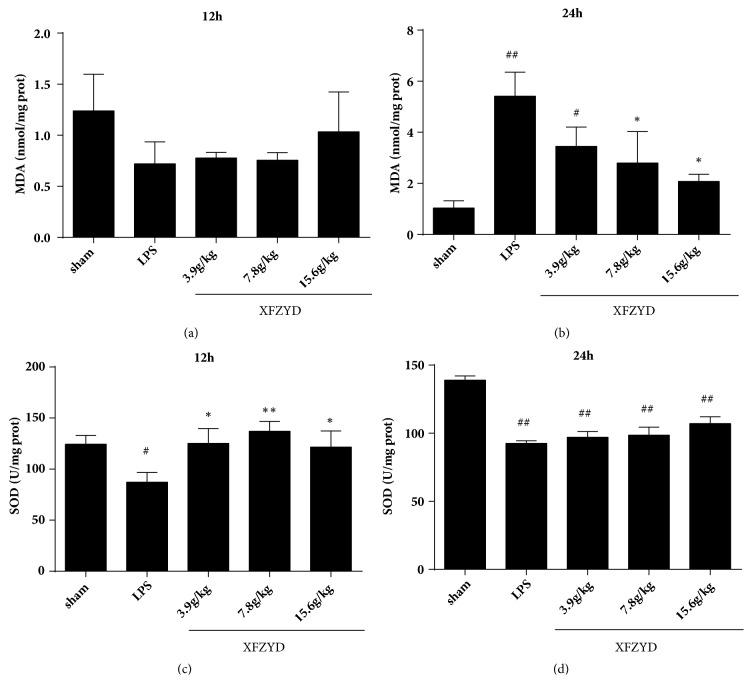
Effects of pretreated with XFZYD on LPS-induced myocardial injury. The superoxide dismutase (SOD) activity was determined by hydroxylamine method. The malondialdehyde (MDA) was detected by thiobarbituric acid (TBA) method. Data are presented as the mean±standard error, n=6 per group. #p<0.05, # p <0.01 versus sham group; *∗*p<0.05, *∗* P <0.01 versus LPS group.

The results showed that there was no difference among the treatment groups for 12 h (Figure 4(a)), while the MDA level in the middle and high dose of XFZYD groups was lower than that in the LPS group (Figure 4(b)). As presented in Figures 3(c) and 3(d), the SOD values were higher than those of the LPS group after 12 h of LPS administration. This displayed that XFZYD has the function of alleviating myocardial injury and has a certain antioxidation effect.

### 3.6. Sepsis-Induced Production of TNF-*α*, IL-1*β*, and IL-6 in Serum or in Heart

**Figure 5:**
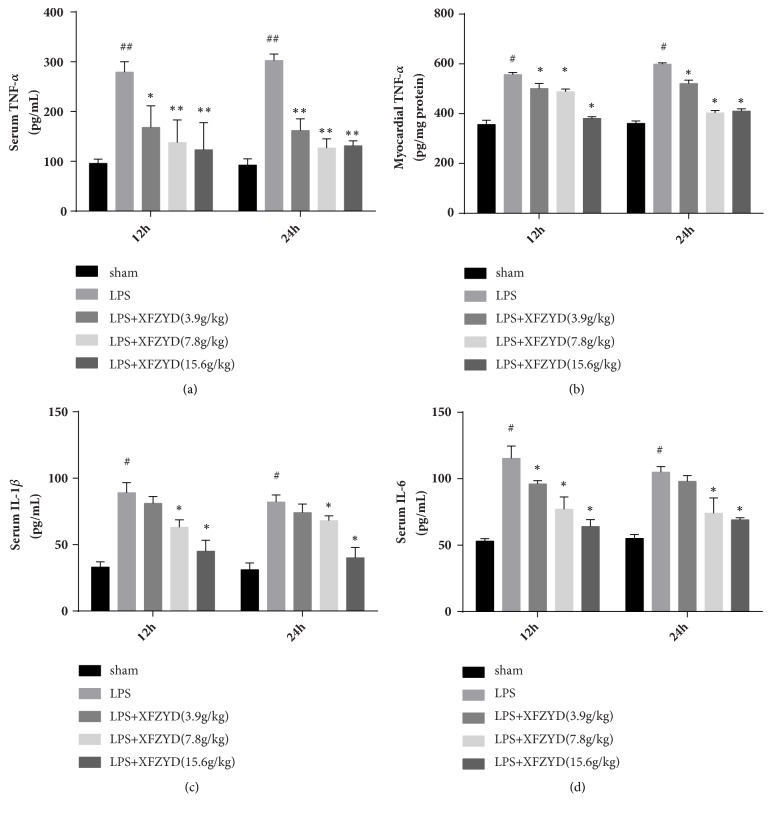
Effects of XFZYD on (a) serum TNF-*α*, (b) myocardial TNF-*α*, (c) IL-1*β*, and (d) IL-6 levels. Data are presented as the mean±standard error, n=6 per group. #p<0.05, # p <0.01 versus sham group; *∗*p<0.05, *∗∗* P <0.01 versus LPS group.

TNF-*α*, IL-1*β*, and IL-6 levels in LPS group at 12 h and 24 h were significantly higher than those in the control group (*p<*0.05). Pretreatment with XFZYD decreased the TNF-*α*, IL-1*β*, and IL-6 levels compared with the rats in LPS model group (Figure 5).

### 3.7. Western Blotting

**Figure 6:**
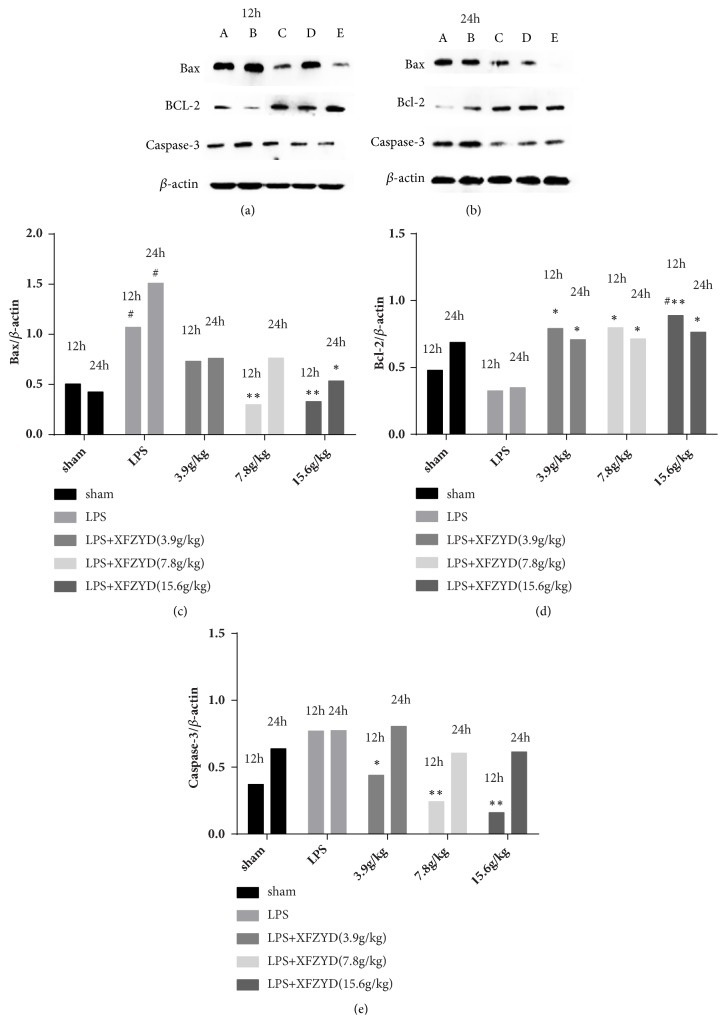
Effect of XFZYD pretreatment on protein expression levels of Bax, Bcl-2, and caspase-3 in myocardial tissue at 12 h and 24 h after LPS administration. Representative images of western blot results are shown as (a) and (b). A: saline; B: LPS; C: LPS+XFZYD (3.9g/kg); D: LPS+XFZYD (7.8g/kg); E: LPS+XFZYD (15.6g/kg). *β*-actin was used as internal control and normalized as 100%. Bars represent mean±SEM values. #p<0.05, # p <0.01 versus sham group; *∗*p<0.05, *∗* P <0.01 versus LPS group.

To investigate the role of XFZYD in myocardial tissue of sepsis rats, the protein expression levels of Bax, Bcl-2, and caspase-3 were assessed by western blot analysis (Figure 6). The LPS group displayed significantly higher Bax expression (P<0.05; Figure 6(c)), lower Bcl-2 expression, and higher caspase-3 expression than the sham group. XFZYD prevented the LPS-induced upregulation or downregulation of Bax and Bcl-2 expression compared with the LPS group at both 12 h and 24 h after LPS administration (P<0.05, Figures 6(c) and 6(d)). By contrast, pretreatment with XFZYD significantly elevated the induction of caspase-3 expression only at 12 h after treating with LPS (p<0.05; Figure 6(e)). This indicates that XFZYD could prevent LPS-induced cardiomyocyte apoptosis by regulating the expression of Bax, Bcl-2, and caspase-3.

### 3.8. Detection of Caspase-3 Activity in Myocardial Tissue of Sepsis Rats

The activity of caspase-3 in cardiomyocytes showed that the level of caspase-3 activity in sham group was low while it increased significantly after 12 hours and 24 hours of administration of LPS. As shown in Table 3, pretreatment with XFZYD significantly reduced the induction of caspase-3 activity by LPS (P<0.05), but it remained significantly higher than that of the sham group (p<0.05). This result indicated that pretreatment with XFZYD may attenuate myocardial apoptosis induced by endotoxaemia.

## 4. Discussion and Conclusion

In our study, a LPS-induced rat model of sepsis was established to mimic human sepsis and used to investigate sepsis-induced cardiac dysfunction and the protective effect of XFZYD. Pretreatment with XFZYD was proved to prevent inflammatory cell infiltration, increase antioxidation activity, suppress myocardial apoptosis, and decrease the levels of TNF-*α*, IL-1*β*, and IL-6 production during sepsis. Also, caspase-3/Bcl-2/Bax signaling pathway was shown to participate in myocardial protection of XFZYD.

Sepsis was defined as “life-threatening organ dysfunction due to a dysregulated host response to infection” [2]. Cardiovascular system, as an important organ system, frequently affected by septic shock and its dysfunction during sepsis have been studied in clinical and basic research for more than 6 decades [18]. Furthermore, patients with presence of cardiac dysfunction in sepsis are at 70-90% risk of death compared with 20% in septic patients without cardiovascular impairment [19]. The mechanisms responsible for sepsis-induced myocardial dysfunction are not clear, but probably via the release of TNF-*α* [20]. The infusion of murine monoclonal anti-TNF antibody into 10 patients with septic shock caused an improvement in cardiac performance [20]. Additionally, TNF-*α* induced caspase activation contributes to the development of cardiac dysfunction [21]. Similarly, IL-1*β* and IL-6 also mediate and regulate inflammatory cytokines. LPS-induced inflammatory stress is reflected in the increase of TNF-*α*, IL-1*β*, and IL-6 and the infiltration of myocardial neutrophils [22]. XFZYD could decrease the levels of IL-1*β* and IL-6 (Figure 5), suggesting that the myocardial protective effect is also related to the anti-inflammatory effect.

The XFZYD is a well-known traditional Chinese medicine for treating cardiovascular diseases. Previous report shows that treatment with XFZYD significantly reduced TNF-*α* and active caspase-3 expression in ischemic stroke [23]. Moreover, XFZYD can improve coagulation dysfunction in sepsis [24]. However, the specific mechanism of XFZYD in improving myocardial dysfunction in sepsis is unclear. In our study, pretreatment with XFZYD improves the cardiac function and attenuates apoptosis and inflammation in septic rats. Also, we found that the expression of caspase-3 and Bax were increased whereas the levels of the two proteins decreased in groups pretreated with XFZYD. This suggests that pretreatment with XFZYD protects against sepsis via inhibition of caspase-3 or Bcl-2/Bax signaling pathway.

In conclusion, the results of the present study demonstrate that XFZYD ameliorates cardiac dysfunction and apoptosis induced by sepsis and suppresses TNF-*α*, IL-1*β*, and IL-6 production and neutrophil accumulation. Additionally, inhibition caspase-3 expression and activity are involved in the protective effects of XFZYD. Thus, the present study provides evidence supporting further investigations into the clinical use of XFZYD in the treatment of sepsis-induced myocardial injury.

## Figures and Tables

**Table 1:** The formulation of XFZYD (one dose).

Herb (Local Name)	Medicinal parts	Amount in application (g)
*Prunus persica* (L) Batch. (Tao Ren)	Seed	12
*Angelicae sinensis (oliv.)* Diels. (Dang Gui)	Root	9
*Ligusticumi chuangxiong* Hort. (Chuang Xiong)	Root	4.5
*Carthamus tinctorius* L. (Hong Hua)	Flower	9
*Paeonia lactiflora* Pall. (Chi Shao)	Root	6
*Rehmannia glutinosa* Libosch.(Di Huang)	Root	9
*Citrus aurantium* L.(Zhi Qiao)	Fruit	6
*Bupleurum chinense *DC. (Chai Hu)	Root	3
*Platycodon grandiflorum* (Jacq) A. DC. (Jie Geng)	Root	4.5
*Achyranthes bidentata* BL. (Niu Xi)	Root	10
*Glycyrrhiza uralensis* Fisch. (Gan Cao)	Root	3

**Table 2:** Effect of XFZYD on mean arterial pressure and heart rate.

Group	mean arterial pressure(mm Hg)		Heart rate(mm Hg)	
	12h	24h	12h	24h
sham	102±2.1	103±1.6	330.8±10.2	332.7±13.2
LPS	81.6±5.8^#^	73.5±4.0^#^	520.0±15.6^#^	541.4±17.3^#^
LPS+XFZYD(3.9g/kg)	82.0±2.4	81.2±2.1	487.6±11.9^*∗*^	503.2±11.5^*∗*^
LPS+XFZYD(7.8g/kg)	87.7±1.8	87.4±3.2	392.0±13.2^*∗*^	426.9±14.6^*∗*^
LPS+XFZYD (15.6g/kg)	91.6±4.5^*∗*^	90.1±3.7^*∗*^	360.5±10.8^*∗*^	354.1±14.3^*∗*^

Results are expressed as mean± SD, n=6; *#p* <0.05 versus normal control group;*∗p *<0.05 versus LPS-insult control group.

**Table 3:** Caspase-3 activity in myocardial tissue of sepsis rats.

Groups	Caspase-3 activity OD_405_ 12h	Caspase-3 activity OD_405_ 24h
Sham	0.31±0.02	0.29±0.02
LPS	0.71±0.03^##^	0.83±0.04^##^
LPS+XFZYD(3.9 g/kg)	0.38±0.04^##*∗∗*^	0.43±0.04^##*∗∗*^
LPS+XFZYD(7.8 g/kg)	0.38±0.03^##*∗∗*^	0.4±0.02^#*∗∗*^
LPS+XFZYD(15.6 g/kg)	0.37±0.05^##*∗∗*^	0.37±0.01^*∗∗*^

Data are given as mean±SEM. #p<0.05, # p <0.01 versus sham group; *∗*p<0.05, *∗* P <0.01 versus LPS group; n=6.

## Data Availability

All data analyzed during this study are included in the manuscript. The raw data used and analyzed during the current study can be available from the corresponding author on reasonable request.

## Abbreviations

XFZYD: Xuefu Zhuyu Decoction
LPS: Lipopolysaccharide
TNF-*α*: Tumor necrosis factor-*α*
MDA: Malondialdehyde
SOD: Superoxide dismutase
ELISA: Enzyme linked immunosorbent assay
IL-1*β*: Interleukin-1*β*
IL-6: Interleukin-6.
